# Wake up and smell the coffee: The potential of faecal volatile organic compounds in paediatric inflammatory bowel disease

**DOI:** 10.1002/ueg2.12618

**Published:** 2024-06-18

**Authors:** Eva Vermeer, Nanne K. H. de Boer, Tim G. J. de Meij

**Affiliations:** ^1^ Department of Paediatric Gastroenterology Emma Children's Hospital Amsterdam University Medical Centre Amsterdam The Netherlands; ^2^ Amsterdam Gastroenterology Endocrinology Metabolism (AGEM) Research Institute Amsterdam University Medical Centre Amsterdam The Netherlands; ^3^ Amsterdam Reproduction & Development (AR&D) Research Institute Amsterdam University Medical Centre Amsterdam The Netherlands; ^4^ Department of Gastroenterology and Hepatology Amsterdam University Medical Centre Amsterdam The Netherlands

**Keywords:** Crohn's disease, IBD, inflammatory bowel disease, ulcerative colitis

In recent years, microbes and their metabolites are increasingly recognised as key players in the pathogenesis of a wide range of gastrointestinal disorders, such as inflammatory bowel disease (IBD), colorectal cancer, irritable bowel syndrome, and coeliac disease, but also diseases outside the gastrointestinal tract.^1^, ^2^ A relatively novel topic in the field of gut metabolomics is the study of faecal volatile organic compounds (VOCs). VOCs are carbon‐based molecules and their chemical composition allows for vaporisation at room temperature. They are therefore released as gases from various matrices, such as blood, urine, or faeces, and are responsible for the odour of a substance. In the gut, these compounds primarily result from the metabolic activities of gut microbiota and the intestinal mucosa.^3^ Alterations in VOC profiles have been described as indicative of various intestinal diseases, including IBD, where specific changes in microbiota composition and diversity, reflected by changes in VOC‐profiles, correlate with disease activity.^4^

Belnour et al. recently conducted a study in a population consisting of 132 case/control pairs of children with IBD and children with gastrointestinal symptoms without IBD.^5^ Their aim was to compare faecal VOC profiles between both groups, and to assess the relation of faecal VOCs with disease phenotype, localisation, severity, and response to treatment. VOCs were analysed through gas chromatography‐mass spectrometry (GC‐MS). They observed significantly decreased mean abundance of 43.6% of 62 measured faecal VOCs in IBD patients compared to controls, which is in accordance with the microbial dysbiosis linked with IBD in literature. Propan‐1‐ol, phenol, and oct‐1‐en‐3‐ol, all being alcohols, were the most distinctive VOCs in children with IBD compared to controls. The first two are products of amino acid degradation (threonine, and tyrosine and tryptophan, respectively) by Enterobacteriaceae and Clostridium species, which are often more abundant in IBD.^6^ The associated amino acids themselves are also linked to gut inflammation.^7^

The gut microbiome and metabolome have gained scientific interest due to the ongoing quest for new non‐invasive biomarkers. Currently, the most used non‐invasive biomarker for the detection and monitoring of IBD is faecal calprotectin (FCP), which is characterised by a high sensitivity for identifying disease activity and luminal inflammation, but suffers from low specificity. The reliance on FCP in clinical practice often leads to unnecessary endoscopies which are especially burdensome for children. Consequently, there is a need for novel more accurate non‐invasive biomarkers. Belnour et al.'s finding that faecal VOCs could differentiate children with IBD from those with gastrointestinal symptoms without IBD, is significant and aligns with recent literature.^8^ However, as the authors note, these findings merely provide insights into the pathogenesis of paediatric IBD, and further research is warranted to develop a clinical biomarker.

Another critical issue in managing IBD is predicting disease severity and response to therapy. In paediatric IBD, therapeutic guidelines regularly recommend a step‐up approach. At baseline, predicting which patients will develop severe disease in the disease course and could benefit from early treatment escalation is challenging. The observation of Belnour et al. of differences in faecal VOC profiles between various states of disease severity and treatment responses is of importance. It highlights the complex pathogenesis of IBD and underscores the need for personalised medical approaches due to the disease's heterogeneity.

Advantages of VOC analysis include its speed and relatively low costs. However, challenges include the influence of environmental factors, especially dietary intake, the lack of standardised sampling, storage and handling protocols, and the variety in used analytical techniques.^9^ GC‐MS is considered the gold standard for VOC analysis,^10^ which separates complex compounds in a GC column, ionises and further separates in the MS column based on mass and transportation time. GC‐MS is highly accurate and can identify specific VOCs on molecular level, but is the most expensive, high‐maintenance and needs trained personnel to use. Electronic noses (eNoses) are more accessible in terms of costs and time. This technique is based on pattern‐recognition of VOC mixtures, allowing for identification of disease‐specific VOC patterns, but cannot measure individual VOCs.

Though the clinical implementation of faecal VOC analysis is still distant, it holds promise as a potential novel non‐invasive biomarker in (paediatric) IBD. Endoscopies will as yet remain necessary for diagnosing IBD to obtain information on disease phenotype, localisation and severity, but faecal VOCs could possibly support to select which patients presenting with gastrointestinal symptoms require endoscopy for suspected IBD diagnosis, given the limited specificity of FCP. Future research should focus on identifying specific VOCs and validating previous results in larger cohorts. The first step however is standardising and refining measurement methodologies to overcome variability in sampling and analysis.

## CONFLICT OF INTEREST STATEMENT

The authors have no conflicts of interest to declare.

## Data Availability

Data sharing is not applicable to this article as no new data were created or analyzed in this study.
